# GeneFriends: gene co-expression databases and tools for humans and model organisms

**DOI:** 10.1093/nar/gkac1031

**Published:** 2022-12-01

**Authors:** Priyanka Raina, Rodrigo Guinea, Kasit Chatsirisupachai, Inês Lopes, Zoya Farooq, Cristina Guinea, Csaba-Attila Solyom, João Pedro de Magalhães

**Affiliations:** Integrative Genomics of Ageing Group, Institute of Life Course and Medical Sciences, University of Liverpool, Liverpool L7 8TX, UK; Integrative Genomics of Ageing Group, Institute of Life Course and Medical Sciences, University of Liverpool, Liverpool L7 8TX, UK; Integrative Genomics of Ageing Group, Institute of Life Course and Medical Sciences, University of Liverpool, Liverpool L7 8TX, UK; Integrative Genomics of Ageing Group, Institute of Life Course and Medical Sciences, University of Liverpool, Liverpool L7 8TX, UK; Integrative Genomics of Ageing Group, Institute of Life Course and Medical Sciences, University of Liverpool, Liverpool L7 8TX, UK; UCAL - Universidad de Ciencias y Artes de América Latina, Faculty of Design, Lima 15026, Perú; Integrative Genomics of Ageing Group, Institute of Life Course and Medical Sciences, University of Liverpool, Liverpool L7 8TX, UK; Integrative Genomics of Ageing Group, Institute of Life Course and Medical Sciences, University of Liverpool, Liverpool L7 8TX, UK; Institute of Inflammation and Ageing, University of Birmingham, Queen Elizabeth Hospital, Mindelsohn Way, Birmingham B15 2WB, UK

## Abstract

Gene co-expression analysis has emerged as a powerful method to provide insights into gene function and regulation. The rapid growth of publicly available RNA-sequencing (RNA-seq) data has created opportunities for researchers to employ this abundant data to help decipher the complexity and biology of genomes. Co-expression networks have proven effective for inferring the relationship between the genes, for gene prioritization and for assigning function to poorly annotated genes based on their co-expressed partners. To facilitate such analyses we created previously an online co-expression tool for humans and mice entitled GeneFriends. To continue providing a valuable tool to the scientific community, we have now updated the GeneFriends database and website. Here, we present the new version of GeneFriends, which includes gene and transcript co-expression networks based on RNA-seq data from 46 475 human and 34 322 mouse samples. The new database also encompasses tissue-specific gene co-expression networks for 20 human and 21 mouse tissues, dataset-specific gene co-expression maps based on TCGA and GTEx projects and gene co-expression networks for additional seven model organisms (fruit fly, zebrafish, worm, rat, yeast, cow and chicken). GeneFriends is freely available at http://www.genefriends.org/.

## INTRODUCTION

The advent of RNA sequencing (RNA-seq) technology has revolutionized biological research (1,2). With RNA-seq we are now able to understand the complexity of transcriptome, which has enabled us to connect the information on our genome with its functional protein expression (3). Moreover, gene co-expression networks provide the potential to identify the gene modules (highly connected sub-networks) that could serve as points for therapeutic interventions (4,5). There are many methods available to cluster the genes in a gene co-expression matrix or map (see the review; (6)). One of the widely used network-based approaches to predict gene functions is the Guilt by association (GBA) method, GBA works on the principle that genes which tend to co-express with each other are functionally related (7,8).

With an increase of >2 million RNA-seq samples in SRA/GEO between 2015 and 2021, the number and power of co-expression databases have also consequently increased. (9–11). To facilitate and promote the usage of co-expression networks, we previously created an online microarray and RNA-seq-based co-expression database, entitled GeneFriends (12,13) for human and mouse genes and for human transcripts. GeneFriends has proven successful for gene prioritization and associating function to poorly annotated genes. Studies employing GeneFriends have focused on diverse topics such as estimating tumorigenic index for cancer initiation and progression (14), genetic analysis for neurological conditions in humans and mice (15), genomics of human metabolic disease (16), development of neuronal subtypes (17), genome evolution (18), genetics of ageing and complex diseases (19,20) and cell senescence (21). Therefore, to keep our tool at the forefront of publicly available co-expression databases we have updated the RNA-seq-based GeneFriends co-expression database for both human and mouse gene and transcript data.

Gene expression and regulation can be highly tissue-specific, and most disease-related genes have tissue-specific expression abnormalities (22,23). Tissue-specific co-expression modules may not be detectable in a co-expression network constructed from multiple tissues or conditions because the correlation signal of the tissue/condition-specific modules is diluted by a lack of correlation in other tissues/conditions (6). To address this need, we have now generated tissue-specific co-expression networks for both humans and mice. Similarly, large-scale RNA-seq data from The Cancer Genome Atlas (TCGA) and Genotype Tissue Expression (GTEx) projects offer a unique opportunity to gain better insight into complex human diseases (24). The co-expression maps generated from these datasets will provide new perspectives about the genes that tend to cluster in a disease setting and help in deciphering the genetic mechanisms underlying various complex diseases, therefore we have now added dataset specific co-expression maps based on RNA-seq data from TCGA and GTEx projects.

Large-scale RNA-seq projects have resulted in rapid generation of transcriptome data for a wide range of organisms (25). In addition to developing a co-expression database for human and mouse samples, we have now created co-expression maps for seven more model organisms (fruit fly, zebrafish, worm, rat, yeast, cow and chicken). The co-expression networks generated from different species will allow users to gain insight on lineage-specific evolution of co-expression networks (26). These multi-species co-expression networks will also give us better understanding of the tissues, pathways and diseases that tend to be conserved or diverged between the species. We believe our latest updated and expanded version of GeneFriends will be useful for a diverse and large number of researchers to understand the complexity, functions and regulation of the genome. GeneFriends is freely available at http://www.genefriends.org

## OVERVIEW OF NEW AND UPDATED GENEFRIENDS CO-EXPRESSION DATABASES

**Figure 1.**
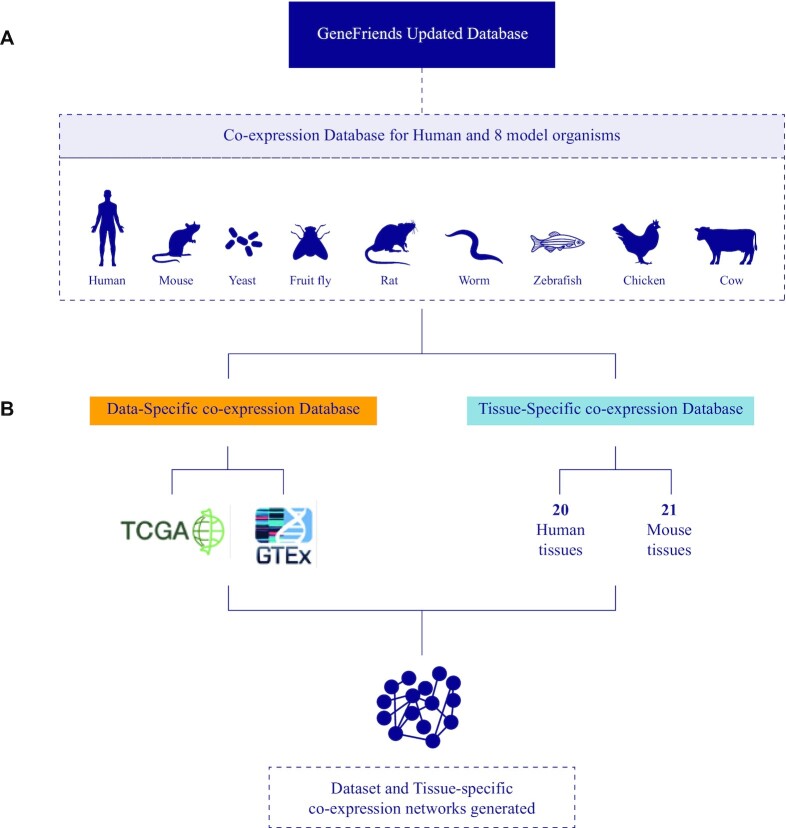
Overview of updated GeneFriends co-expression database. (**A**) Species for which co-expression networks are available. (**B**) Details for dataset and tissue-specific co-expression databases. The read counts for creating human RNAseq co-expression maps (bulk RNAseq, tissue-specific, TCGA, GTEx) were downloaded from *recount2* database. The read counts for both bulk and tissue-specific co-expression maps based on model organisms (mouse, fruit fly, zebrafish, worm, rat, yeast, cow and chicken) were obtained from ARCHS^4^ database.

In addition to updating the previous GeneFriends co-expression database for human genes and transcripts (van Dam et al. 2015), we have now added RNA-seq based co-expression database for (a) mouse genes and transcripts; (b) fruit fly, zebrafish, worm, rat, yeast, cow and chicken genes; (c) TCGA project genes; (d) GTEx project genes; (e) tissue-specific co-expression maps for human genes; (f) tissue-specific co-expression maps for mouse genes (Figure 1).

### Human and mouse co-expression gene and transcript co-expression database

The new human and mouse co-expression databases were constructed from 46 475 and 34 322 RNA-seq samples, respectively. The updated GeneFriends database contains co-expression data for 44 896 human genes and 31 236 mouse genes. The transcript co-expression data comprises of 145 455 human transcripts and 66 327 mouse transcripts. The biotype of genes and transcripts for both human and mouse data is given in Table 1. One of the unique features of GeneFriends co-expression database are its co-expression maps for non-coding genes like long non-coding RNA (lncRNA) and microRNA (miRNA) which can be useful in providing the insights for regulatory mechanism of gene expression at both transcriptional and post-transcriptional level. The updated GeneFriends databases have co-expression data for nearly 16 450 human and 6436 mouse non-coding genes.

**Table 1.** The biotype of genes/transcripts in updated GeneFriends co-expression databases

**Co-expression database**	**Number of protein coding genes**	**Number of non-coding genes (%)**	**Others (%)**
Human genes	19 642 (43.8%)	16 450 (36.6%)	8804 (19.6%)
(*n*=44 896)			
Human transcripts	89 433 (61.5%)	39 776 (27.3%)	16 246 (11.2%)
(*n*=145 455)			
Mouse genes	19 715 (63.1%)	6436 (20.6%)	5085 (16.3%)
(*n*=31 236)			
Mouse transcripts	42 852 (64.6%)	12 928 (19.5%)	10 547 (15.9%)
(*n*=66 327)			
Fruit fly genes	12 165 (85.6%)	1844 (13.0%)	204 (1.4%)
(*n*=14 213)			
Zebrafish genes	25 740 (85.6%)	4029 (13.4%)	304 (1.0%)
(*n*=30 073)			
Worm genes	18 463 (87.3%)	1266 (6.0%)	1424 (6.7%)
(*n*=21 153)			
Rat genes	17 685 (81.5%)	3023 (13.9%)	991 (4.6%)
(*n*=21 699)			
Yeast genes	6015 (89.8%)	616 (9.2%)	68 (1.0%)
(*n*=6699)			
Cow genes	17 019 (85.1%)	2582 (12.9%)	396 (2.0%)
(*n*=19 997)			
Chicken genes	15 255 (86.0%)	2210 (12.5%)	264 (1.5%)
(*n*=17 729)			
TCGA genes	19 164 (42.6%)	13 400 (29.8%)	12 434 (27.6%)
(*n*=44 998)			
GTEx genes	19 262 (42.8%)	14 016 (31.2%)	11 695 (26.0%)
(*n*=44973)			

*n= total number of genes present in the co-expression database, others = pseudogenes, TR (T-cell receptor genes), IG gene (immunoglobulin genes).

^#^Read counts for human RNAseq based co-expression maps were downloaded from *recount2* database and read counts for model organisms were obtained from ARCHS^4^ database.

We have also compared the top 5% of ten randomly selected human genes and their co-expression partners, which are present in both previous version (13) and new updated version of GeneFriends (Supplementary Table S1). The percentage of the average overlap between the ten genes was 30.5% with a standard deviation of 4.97%. This difference between the two versions could be due to the difference in number of samples. The previous version was constructed from only 4133 RNA-seq samples as compared to the updated version, which is based on 46 475 samples. However, when we compared the functional enrichment of the top 5% co-expressed partners for some of these genes, the overlap was stronger suggesting that although the overlap between the co-expressed partners was low but overall they were associated with similar functional categories (Supplementary Data S1).

### Model organisms’ gene co-expression database

Apart from mouse gene and transcript co-expression maps, we also constructed gene-co-expression maps for seven more model organisms. *Drosophila melanogaster* (number of samples = 9924), *Caenorhabditis elegans* (number of samples = 2935), *Danio rerio* (number of samples = 4004), *Rattus norvegicus* (number of samples = 3373), *Saccharomyces cerevisiae* (number of samples = 3268), *Bos taurus* (number of samples = 2039) and *Gallus gallus* (number of samples = 1649). The number and biotype of genes used to create the co-expression networks for model organisms are given in Table 1. The one-to-one orthologs between different species is represented in Supplementary Table S2.

### Dataset-specific gene co-expression database (TCGA and GTEx)

The TCGA co-expression map is constructed from 10 544 RNA-seq samples encompassing samples from 33 cancer types. The GTEx co-expression database is based on 9662 RNA-seq samples from 31 tissues. The details of the cancer types and tissue distribution for GTEx and TCGA data is given in Supplementary Figure S1. TCGA and GTEx co-expression databases contains data for 44 998 and 44 973 genes, respectively. The detailed information about the biotype of the genes is given in Table 1.

### Tissue-specific gene co-expression database (human and mouse)

The human tissue-specific co-expression maps were generated for 20 tissues from 46,080 RNA-seq samples. In the case of the mouse, 53,098 RNA-seq samples were used to generate 21 tissue-specific co-expression maps. The number of samples used to create each tissue-specific co-expression map for the human and mouse databases is given in Supplementary Figure S2. For each tissue co-expression map, the number of genes were filtered on the basis of their expression by excluding genes that were not expressed in at least 20% of the samples (Supplementary Table S3). The list of top 100 co-expressed genes for each tissue was determined by calculating the median of correlation values for each gene with respect to its co-expressed partners across the database (Supplementary Data S2). The distribution of median correlation coefficients for genes among different tissues in human and mouse tissue-specific co-expression database is given in Supplementary Figure S3.

## GENEFRIENDS GENE AND TRANSCRIPT DATA COMPARISON

**Figure 2.**
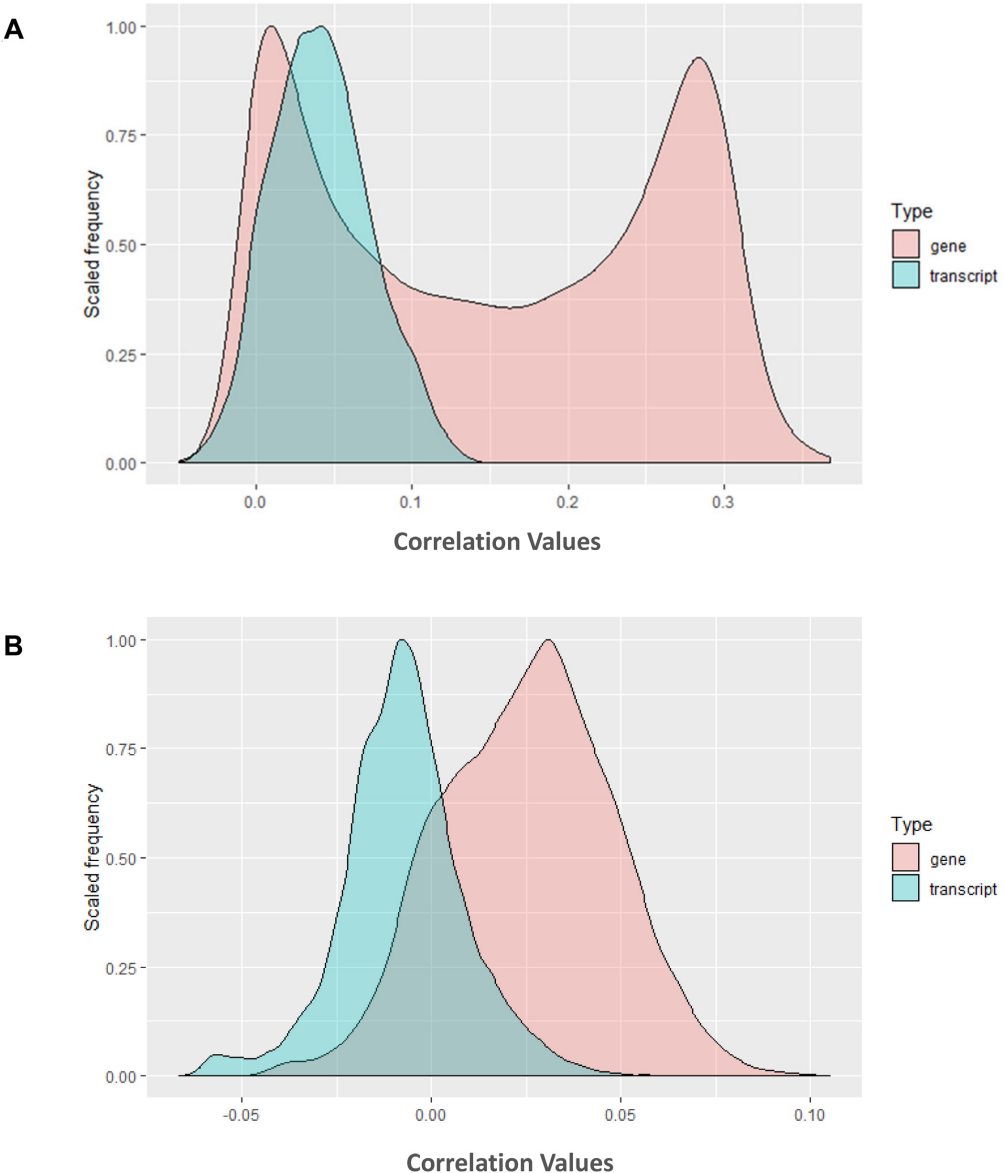
(**A**) Distribution of correlation coefficient values between human genes and transcripts. (**B**) Distribution of correlation coefficient values between mouse genes and transcripts.

To explore the differences between the gene and transcript co-expression maps in the human and mouse co-expression databases, we compared the median of Pearson correlation coefficient values for each gene/transcript with respect to its co-expression partners across the GeneFriends database. For transcripts, the median of different transcripts of the same gene was calculated for doing comparison. A total of 34 920 human and 25 459 mouse genes and its transcripts were analysed. 78% of human and 70% of mouse genes had more than one transcript. While comparing the co-expression maps of human genes and transcripts, the overall co-expression values of genes were significantly higher than the co-expression values of transcripts (Figure 2A). The range of Pearson's correlation coefficient values was widely distributed in genes encompassing both positive and negative values (Figure 2A). However, transcripts had smaller positive correlation coefficient values than genes. This observation could be due to the fact that the transcript values are the median of different transcripts of the gene and different transcripts of the same gene may have different trends of correlation coefficient values. Similar trends were observed for the mouse co-expression database, where mouse genes had higher correlation coefficients than transcripts (Figure 2B), although the range of correlation coefficients were not as widely distributed as in humans (Figure 2A). These results indicated that different transcripts arising from the same gene are often expressed under different conditions and are most likely to play different roles in different processes or sometimes these transcripts may even be non-functional (27).

## PATHWAY ANALYSIS IN GENEFRIENDS

**Figure 3.**
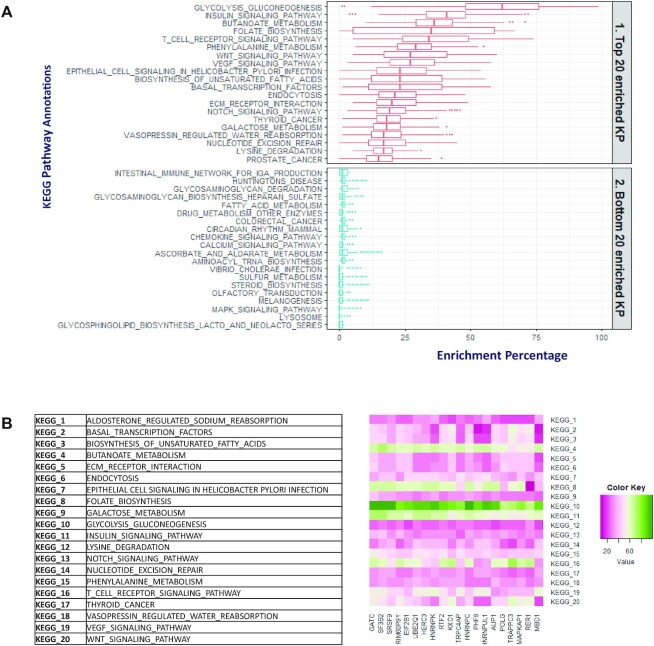
KEGG pathway enrichment analysis among the GeneFriends human database genes. (**A**) Top 20 and bottom 20 KEGG pathway annotations among the top 5% of GeneFriends human genes and their co-expressed partners. (**B**) KEGG pathway annotations among the top 20 GeneFriends genes with maximum number of KEGG pathway enrichments. The colour of the heat map represents the range of KEGG pathway enrichments among these 20 genes, pink = low number of KEGG pathway enrichments and green = high number of KEGG pathway enrichments.

Since the primary purpose of the co-expression database is to determine the function of the co-expressed genes, we investigated the KEGG pathway genes to assess the consistency of the co-expression data with pathway annotations. We compared the number of enriched KEGG pathway genes between top and bottom 5% of co-expressed genes in human GeneFriends co-expression database. A total of 186 KEGG pathway gene sets from Molecular Signatures Database (MSigDB) v7.0 were analysed. The top 5% of co-expressed genes had significantly higher number [Median, Interquartile range (IQR) = 107(50 – 280)] of KEGG pathway enrichments in comparison to bottom 5% [median (IQR) = 6(6 – 203)] (Supplementary Figure S4). This was followed by further analysing the top 5% of co-expressed genes with most enriched KEGG pathway genes for each 186 KEGG pathway gene sets (Supplementary Figure S5) and comparing top 20 and bottom 20 KEGG pathway annotations among the human GeneFriends co-expression database (Figure 3A). The KEGG pathway enrichments like Glycolysis, insulin signalling, folate synthesis and WNT signalling were among the top 20 enriched KEGG pathway annotations. These top 20 KEGG pathway annotations were related to metabolic pathways, DNA repair and signalling. The pathway annotations present in bottom 20 were associated with immune system and infection (Figure 3A). After this, we selected the top 20 genes from the GeneFriends database with maximum number of KEGG pathway annotations, and checked which pathways are most enriched in these top 20 genes (Figure 3B). Here also we observed that the pathways related to metabolism and cell signalling were among the top enriched KEGG pathways annotations. All these observations from KEGG pathway analysis indicated that genes that are enriched in KEGG pathway often tend to co-express together, underscoring that genes that are co-expressed tend to work cooperatively in the same biological pathways.

## VALIDATION OF GENEFRIENDS DATA

**Figure 4.**
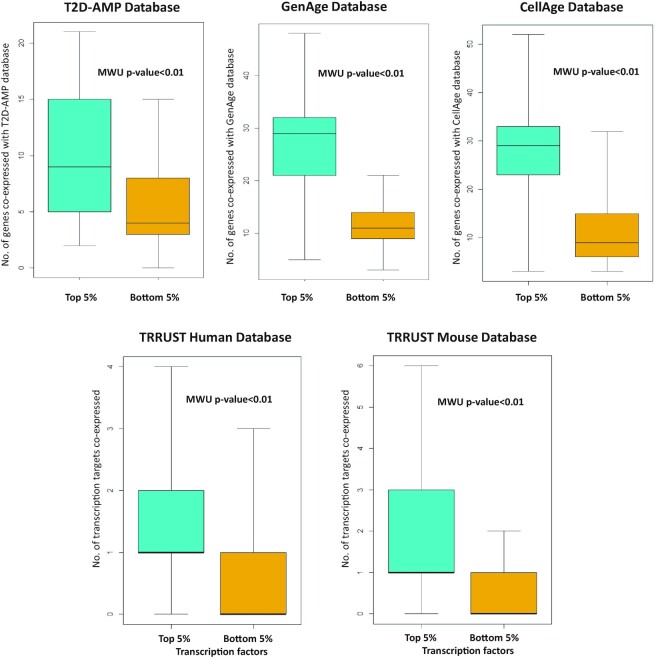
Comparing top and bottom 5% co-expressed gene partners of T2D-AMP, GenAge, CellAge and TRRUST database genes.

To assess the quality of the GeneFriends co-expression database we compared the top and bottom 5% of the genes that are present in some widely used databases. Genes from databases such as GenAge (28), CellAge (21), T2D-AMP Knowledge Portal and TRRUST (29) and their co-expressed partners were analysed to ascertain whether or not the genes that are linked to some diseases or processes tend to co-express together (Figure 4). GenAge is a curated database of genes related to ageing (28). We analysed co-expression data of 298 GenAge genes. The top 5% of GenAge genes present in GeneFriends database had significantly higher number of GenAge genes as their co-expressed partners as compared to the bottom 5% [median(IQR): top = 29(21–32); bottom = 11(9–14)]. Similar trend was observed for 272 CellAge database (a curated database of cell senescence genes) and their co-expressed partners, where top 5% had significantly higher number of CellAge genes co-expressed in comparison to bottom 5% [median(IQR): top = 29(23–33); bottom = 9(6–15)]. We were also interested to see how often genes that are related to some diseases may co-express with each other. To investigate this we analysed 132 type 2 diabetes (T2D) effector genes from the T2D-AMP database (https://t2d.hugeamp.org/effectorgenes.html). We observed that T2D effector genes co-express with each other as the top 5% had a significantly higher number of T2D genes with respect to the bottom 5% [median(IQR): top = 9(5–15); bottom = 4(3–8)].

To further validate our observations we also tested transcription factors and their targets from TRRUST database version 2 (29). TRRUST database is a manually curated database of human and mouse transcriptional regulatory networks. As genes that co-express with each other may also help in co-regulating each other, hence we postulated that transcription targets should co-express with their respective transcription factors. We removed transcription factors where the relationship with the target was unknown. For the human co-expression database, 603 human transcription factors were analysed. These transcription factors were then matched with 1710 transcriptional targets. The top 5% of co-expressed genes of all transcription factors had a significantly higher number of transcriptional targets expressed in comparison to bottom 5% [median(IQR): top = 1(1–2); bottom = 0(0–1)]. A total of 223 transcription factors had at least one transcriptional target present in the top 5% co-expressed genes. In the case of the mouse co-expression database, co-expression data for 703 mouse transcription factors were checked for 2100 transcriptional targets. Similarly for human transcription factors, top 5% mouse co-expression partners of transcription factors had a significantly higher number of transcriptional targets in comparison to bottom 5% [median(IQR): top = 1(1–3); bottom = 0(0–1)]. A total of 317 transcription factors had at least one transcriptional target present in the top 5%. All these observations indicated that GeneFriends co-expression database is successfully able to identify the genes that are co-expressed and co-regulated together.

## GENERATING TAU BASED TISSUE-SPECIFIC GENES

Apart from generating tissue-specific co-expression maps for human and mouse data, we also created tau-based tissue-specific gene sets for RNA-seq data downloaded from the SRA database. A Tau (τ) tissue specificity index was calculated for each gene for every tissue. A τ index was used as an indicator to check how tissue specific or broadly expressed a gene is, with a τ of 1 indicating expression specific to only one tissue, and a τ of 0 indicating equal expression across all tissues (30). We used a τ value of 0.8 as cut-off to create our τ based tissue-specific database. The number of tissue-specific genes for each tissue were created for human and mouse data (Supplementary Table S4). Tissue-specific gene lists were generated for 20 human and 21 mouse tissues (Supplementary Data S3).

## COMPARISON OF HUMAN AND MOUSE CO-EXPRESSION NETWORKS

**Figure 5.**
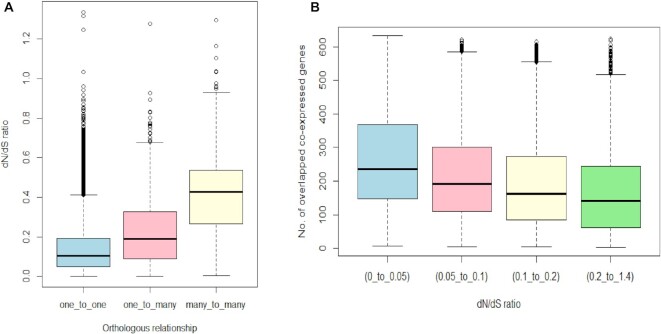
(**A**) Comparison of d*N*/d*S* values of homologs with three different relationships (one to one, one to many and many to many). The Mann–Whitney test showed significant difference between all three comparisons (one to one versus one to many, one to many versus many to many and one to one versus many to many). (**B**) Comparison of the d*N*/d*S* values between the top 5% of human and mouse co-expression gene networks.

We analysed human and mouse co-expression networks from an updated GeneFriends co-expression database to decipher the evolutionary differences and similarities between human and mouse co-expression maps. We compared 24 434 genes that have a homolog in both human and mouse gene co-expression databases. In our co-expression database, 14,911 genes were one-to-one orthologs, while the remaining mouse and human homologs had a one-to-many or many-to-many relationship. To understand the impact of duplication events on the divergence of humans and mice, we compared the d*N*/d*S* ratios of homologous genes with different types of homology (Figure 5A). The one-to-one orthologs had the lowest d*N*/d*S* ratio as compared to the many to many, which had the highest d*N*/d*S* ratio. Next, we compared 14 911 one to one orthologs among the top 5% of co-expressed genes. The d*N*/d*S* ratio values were divided into four groups to check how the increase/decrease in these values may relate to overlapping between two co-expression networks (Figure 5B). We observed that the group with the lowest d*N*/d*S* values had the highest number of overlapped co-expressed genes. This supported the hypothesis that non-synonymous substitutions influence the conservation of co-expression connectivity (31). Therefore, the higher the number of non-synonymous substitutions, the less conserved is a co-expression network.

## COMPARISON BETWEEN GTEx AND TCGA CO-EXPRESSION NETWORKS

**Figure 6.**
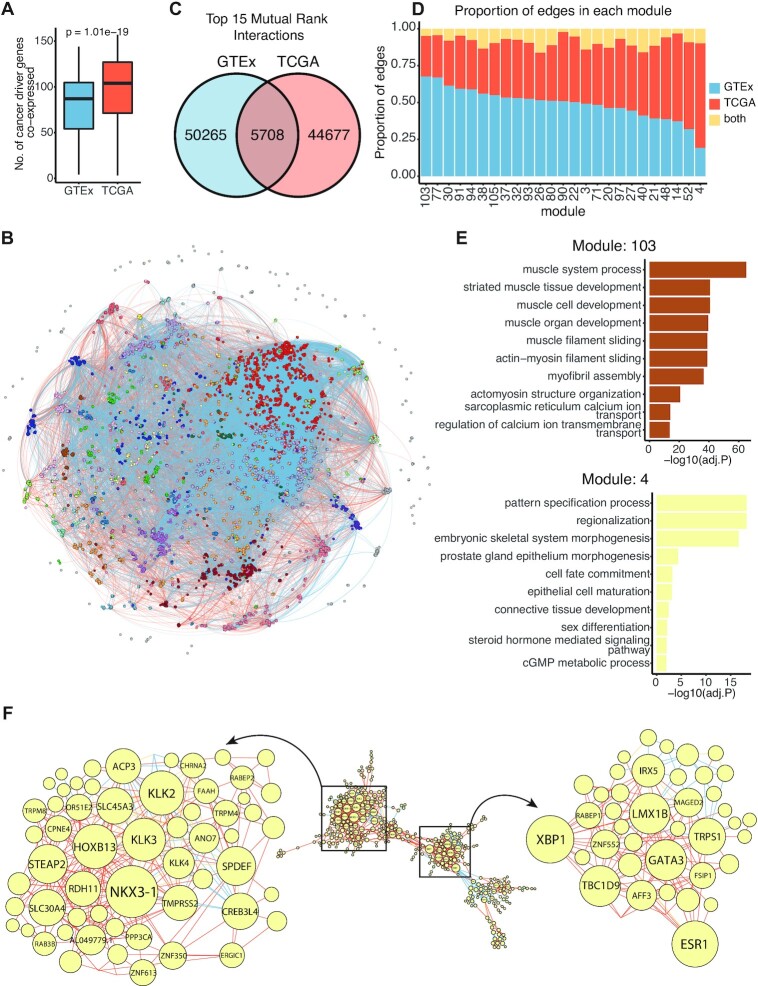
Comparison between GTEx and TCGA co-expression networks. (**A**) Boxplots showing the number of cancer driver genes in the top 5% co-expression partners of a cancer driver gene. The middle bar of the boxplot is the median. The statistical significance (p-value) was calculated using a two-sided Wilcoxon rank-sum test. The box represents the interquartile range (IQR), 25–75th percentile. Whiskers represent a distance of 1.5× IQR. (**B**) A unified network comprised edges from the top connections (mutual rank < 15) of GTEx and TCGA co-expression networks. Circles (nodes) represent protein-coding genes. Circle colours correspond to modules. Lines (edges) represent co-expression between two protein-coding genes. Edge colours represent types of edge (blue: GTEx only, orange: TCGA only, yellow: both GTEx and TCGA). (**C**) Overlap between edges from GTEx network and TCGA network. (**D**) The proportion of edges in each module. The bar chart represents the proportion of edges in each module by GTEx only (blue), TCGA only (orange) and both GTEx and TCGA (yellow). (**E**) Top 10 Gene Ontology (GO) terms related to genes in module 103 and module 4. (**F**) Network representation of module 4. Circles (nodes) represent protein-coding genes in module 4. Circle size corresponds to the number of connections of the circle (degree). Lines (edges) represent co-expression between two protein-coding genes and are coloured by types of edge (blue: GTEx only, orange: TCGA only, yellow: both GTEx and TCGA).

Because the GTEx co-expression network was derived from only non-cancerous tissues, whereas the TCGA co-expression network was constructed from neoplasms, we were interested in comparing these two networks. We first compared the top 5% co-expressed partners of 562 cancer driver genes (32). TCGA network presented a significantly higher number of cancer driver genes in the top 5% co-expression partners of a cancer driver gene than in GTEx network [median (IQR): GTEx = 87 (54–104.75); TCGA = 104 (71.25–127)] (Figure 6A), suggesting that cancer driver genes were more often co-expressed with each in the cancerous tissues than the non-cancerous tissues. We next selected very top connections between protein-coding genes (mutual rank < 15) from GTEx (nodes = 16 825; edges = 55 973) and TCGA (nodes = 16 097; edges = 50 385) networks and combined these connections to construct a unified network containing 18 475 protein-coding genes and 100 650 connections (Figure 6B, Supplementary Data S4a). We found that only 5708 connections were shared between GTEx and TCGA networks (Figure 6C), while 50 265 and 44 677 connections were unique to the GTEx (blue lines in Figure 6B) and TCGA (red lines in Figure 6B) networks, respectively. This result indicates that the very top co-expression partners between genes in cancer and normal tissues were different.

We detected 111 modules (clusters) with 25 modules containing >150 genes using a multi-level optimisation algorithm (Supplementary Data S4b). Out of these 25 modules, we found modules mainly consisting of GTEx edges and those enriched in TCGA edges (Figure 6D). For instance, 68% of edges in module 103 were from GTEx network (Figure 6D), this module enriched in genes related to muscle functions **(**Figure 6E top, Supplementary Data S4c). On the other hand, edges from the TCGA network contributed to 71% of edges in module 4 (Figure 6D). This module was enriched in developmental processes (Figure 6E bottom, Supplementary Data S4c), consistent with the idea of the reactivation of developmental pathways in cancer initiation and progression. Hub genes of module 4 included prostate cancer-related genes such as *NKX3-1* (33), *KLK2* (34), *KLK3* (35) and *HOXB13* (36) (Figure 6F, left). Furthermore, we also noticed several genes implicated in breast cancer, such as *ESR1* (37), *GATA3* (38), *XBP1* (39), *TBC1D9* (40) and *TRPS1* (41) (Figure 6F, right).

We further identified gene modules in which cancer driver genes are enriched. Interestingly, cancer driver genes significantly overrepresented in module 32 (OR = 1.92; adj. *P*-value = 3.9 × 10^−4^), which relate to immune system functions, and module 90 (OR = 1.73; adj. *P*-value = 1.6 × 10^−6^), which is associated with RNA processes (Supplementary Figure S6 A–C, Supplementary Data S4d). Interestingly, both modules did not have a bias toward TCGA or GTEx connections (module 32: 53% GTEx edges, 40% TCGA edges; module 90: 51% GTEx edges, 47% TCGA edges) (Figure 6D). Therefore, while the connections between cancer driver genes were more pronounced in the TCGA network (Figure 6A), cancer driver genes are not exclusively located within the module with mostly cancer-only connections.

Taken together, by integrating co-expression networks from non-cancerous tissues and tumours, we were able to identify gene modules that are co-expressed exclusively in cancer. These results confirm our GTEx and TCGA co-expression networks' reliability and highlight the differences between gene networks in normal and cancer tissues. We expected that our GTEx and TCGA co-expression networks would lead to the identification of novel cancer-related genes, which will serve as potential biomarkers or therapeutic targets.

## GENEFRIENDS WEB SERVER

**Figure 7.**
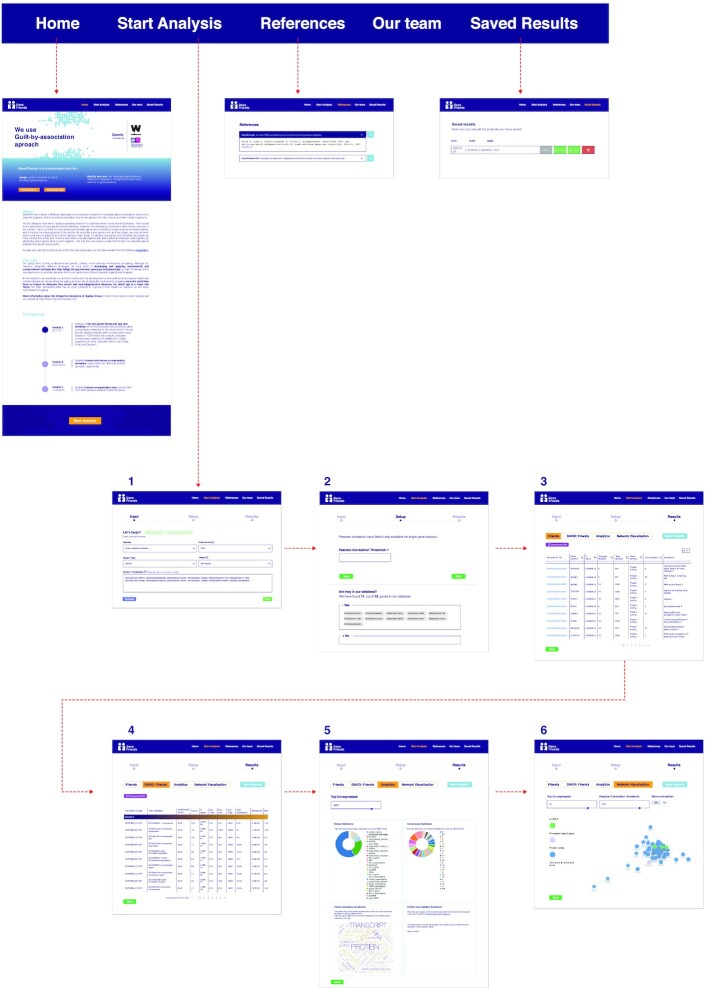
A graphical overview of the steps involved in retrieving results from GeneFriends: (1) input genes, (2) set up Pearson correlation threshold values, (3) top co-expressed genes as output, (4) functional enrichment of top 5% co-expressed genes via DAVID API, (5) analytics of the top co-expressed genes, (6) visualization of the network of co-expressed genes.

The new GeneFriends website is more intuitive and faster with easy data accessibility (Figure 7). The first step is to input one or multiple gene/transcript ID’s. The second step involves selecting species (Human, Mouse, Fruit fly, Zebrafish, Worm, Rat, Yeast, Cow and Chicken), data source (SRA, TCGA, GTEx) and tissue of interest (User can select all tissues together, if not interested in tissue specific co-expression). The results section contains the list of the top co-expressed genes, top functional enrichment categories of the co-expressed list of genes using DAVID API, Analytics and Network Visualization. We refer readers to the Supplementary section (GeneFriends Web Application Tutorials, Supplementary Figures S7–S12) for a detailed tutorial and usage guide of GeneFriends.

## FUTURE PLANS

To better serve the research community, in the future we aim to expand GeneFriends to include more features and functionalities. Although GeneFriends provides tissue-specific co-expression networks based on the SRA database, our current TCGA and GTEx co-expression networks are not tissue-specific, and we intend to include the tissue-specific networks for TCGA and GTEx in future versions. We also plan to add cancer-specific co-expression networks for the users interested in comparing networks generated from various cancer-types. Because of the lack of gender-related metadata for many samples, our GeneFriends database does not have gender-specific co-expression maps, however, accumulating evidence suggests that gender has an impact on gene expression in various tissues (42,43); therefore we aim to curate our samples and create gender-specific co-expression maps in future updated versions of the database. Finally, we aim to generate conserved co-expression network to compare network from different species.

## MATERIALS AND METHODS

### Generation of co-expression database

Human RNA-seq read counts for 46 475 samples were downloaded from the *recount2* database (44). Human gene expression data was downloaded with recount Bioconductor package (version 1.22.0) (44) and transcript data was downloaded with recountNNLS R package (version 0.99.7) (45) Mouse RNA-seq based read counts were obtained for 34 322 samples from ARCHS^4^ database with rhdf5 Bioconductor R package (version 2.40.0) (46). The human samples were aligned against the GRCh38 human reference genome, and mouse samples against the GRCm38 mouse reference genome. The reads were then normalized by dividing the expression per gene/transcript to the combined expression of all genes/transcripts per sample. In addition to human and mouse, co-expression maps for fruit fly, zebrafish, worm, rat, yeast, cow and chicken were also created from read counts downloaded from ARCHS^4^ database with rhdf5 Bioconductor R package (version 2.40.0).

To create co-expression maps, we used weighted Pearson correlation method (13). This was followed by constructing mutual rank maps by employing the same approach used in COXPRESdb (11). We used guilt by association method to create co-expression networks. The genes that were not expressed in at least 20% of the samples were excluded from the database. The biotype of genes and transcripts for both human and mouse data was identified using biomaRt (version 2.46.3).

Tissue-specific co-expression maps were also created for both human and mouse data. For human tissue-specific co-expression maps, read counts were downloaded from *recount2* database (44) for 20 tissues from 46 080 RNA-seq samples. Mouse tissue-specific co-expression database comprised of 21 tissues based on 53098 samples. The read counts were downloaded from ARCHS^4^ database (46). The low expressed genes were filtered out from the analysis by keeping only genes that were expressed in at least 20% of samples. There is an overlap between the RNAseq samples used for creating bulk and tissue-specific human and mouse co-expression maps. The larger number of samples in tissue-specific co-expression maps is due to the addition of more samples in the respective databases in a period of time.

TCGA (number of samples = 10 544) and GTEx (number of samples = 9662) co-expression databases were also created by using raw read count from *recount2* database (44). The samples included in TCGA and GTEx co-expression databases were excluded from the human co-expression database. The reason for excluding TCGA samples was to avoid any bias in the co-expression database moreover; cancer-related samples do not generalize well with overall human co-expression networks (47). The GTEx samples were excluded to observe the difference between the tau-based tissue-specific database created from SRA data with respect to the database created from GTEx data by Palmer *et al.* (48).

### Construction of tau-based tissue-specific database

Tau (τ) based tissue-specific genes database was created for 20 human and 21 mouse tissues. The read counts were obtained from *recount2* and ARCHS^4^ database. The read counts for each gene among all tissues were then converted to transcripts per million (TPM) values. This was followed by calculating the mean TPM value for each gene per tissue. The mean TPM values were then log transformed. These used values were then used to create a τ index for each gene. A τ of 1, indicated that expression is specific only to one tissue, and a τ of 0 indicated equal expression across all tissues (30).

### Functional and pathway analysis

We used WebGestalt 2019 (49) to do the Overrepresentation Enrichment Analysis for each of the gene ontology categories (Biological Process. Cellular Component and Molecular Function). The significance level was determined at FDR <0.05 and the multiple test adjustment was done using the Benjamini–Hochberg method. We verified our enrichment results by repeating the analysis using DAVID’s annotation clustering (50). P-value and FDR < 0.05 were considered significant. We also used ClusterProfiler Version 3.14.3 (51) to visualize the GO terms (FDR < 0.05) obtained from DAVID. For KEGG annotation analysis (52), genes lists with their enriched KEGG pathway annotations were obtained from the KEGG subset of canonical pathways (CP) from Molecular Signature Database Version 7.0 (53–55). The box plot and heat map for KEGG pathway analysis were created using R (version 4.0).

### Evolution-based analysis

To identify any differences in the evolutionary conservation of genes present in human and mouse co-expression networks we performed d*N*/d*S* analysis. The d*N*/d*S* values were obtained using biomaRt R package release 96 (version 2.46.3).

### Comparison of GTEx and TCGA database

We obtained a list of 568 cancer driver genes from IntOGen (32). We converted gene symbols to ensemble IDs using Ensembl database (version 102) (56) implemented in the biomaRt R package (version 2.46.3) (57), resulting in 562 cancer driver genes in total. We extracted the top 5% co-expression partners of each cancer driver gene from GTEx network (total = 44 999 genes) and TCGA network (total = 44 972 genes) separately. Thus, for each cancer driver gene, the top 5% co-expression genes were 2250 and 2249, respectively, in GTEx and TCGA. We compared the number of cancer driver genes presented in the top 5% co-expressed genes of each cancer driver gene between GTEx and TCGA using a two-sided Wilcoxon rank-sum test.

We extracted the top co-expressed genes by mutual rank <15 for GTEx and TCGA networks separately. We further kept only connections between protein-coding genes identified using Ensembl database (version 102). The top connections from GTEx network consisted of 16 825 protein-coding genes (nodes) and 55 973 connections (edges), while those from TCGA network comprised 16 097 nodes and 50 385 edges. We next combined these top connections from both networks to construct a unified network containing 18 475 nodes and 100 650 edges. We then classified edges by the network of origin as edges from GTEx network, edges from TCGA network, and edges from both GTEx and TCGA networks. Network module detection was performed using the multi-level optimisation algorithm (58) implemented in the igraph R package (version 1.2.6) (59). Gephi (version 0.9.2) was used for network visualisation. We next extracted modules with more than 150 genes and performed Gene Ontology (GO) enrichment analysis for genes in each module using the clusterProfiler R package (version 3.18.1). All genes in the network were used as a background.

### Statistical analysis

Mann–Whitney *U* tests was used to test the significance between the correlation coefficients among top 5% and bottom 5% co-expressed partners of genes and to compare the distribution of d*N*/d*S* scores between the human and mouse co-expression database. The median and interquartile ranges (IQR) were calculated by R package (version 4.0). For comparing GTEx and TCGA co-expression networks, multiple-hypothesis testing correction was done using Benjamini–Hochberg procedure. Biological processes with adjusted *P*-value <0.05 were considered significantly enriched GO terms. The enrichment of cancer driver genes within each module were tested using Fisher's exact test.

### GeneFriends webserver

The new version of GeneFriends has been developed using Vue.js 3 as view engine in the frontend and Node.js in the backend. Since our data is inherently graph-like in form, and since speed is only required for data fetching, the analytical database Neo4j was chosen. Also, the styles library PrimeVue, together with vanilla CSS, was used to implement structure and appearance in the frontend. Finally, the frontend, the backend, and the database are within their own Docker container. In order to communicate with the third party DAVID API, a Python 2.7 module was used within the backend Docker container.

## CONCLUSIONS

Large-scale gene co-expression networks have proven effective for analysing and discovering new gene functions and associations (60). There are several other online databases and tools based on co-expression data, as this is a very timely and widely used approach. Examples of tools based on co-expression data derived from public databases are COXPRESdb (11), iNETModels (61) and CoCoCoNET (47). The features that make GeneFriends unique and exceptional are its transcript-based co-expression maps and inclusion of co-expression networks for non-coding genes. In comparison to other publicly available co-expression databases, which focus more on protein coding genes, our GeneFriends database encompasses co-expression networks for about 16 000 and 6000 non-coding genes for, respectively, humans and mice. The transcript co-expression data comprises 145 455 human transcripts and 66 327 mouse transcripts. These transcripts and non-coding gene data based co-expression networks are crucial in providing novel insights for different splice variants and non-coding genes, such as miRNAs and lincRNAs. Understanding the regulated and coordinated changes that occur between non-coding RNA and coding (including splice variants) gene expression may reveal novel important players in many biological processes and diseases. Since different splice variants from the same gene can have different functions, measuring the differential expression of all splice variants together can result in misleading conclusions. GeneFriends allows putative functions to be assigned to each splice variant and non-coding genes.

Furthermore, we validated GeneFriends with genes from GenAge (28), CellAge (21), T2D-AMP Knowledge Portal and TRRUST database (29). Our validation results especially using a curated transcription factor-transcriptional target database show that genes that are co-expressed with each other also tend to co-regulate each other. In addition, in our new version, we have included tissue-based and dataset specific co-expression maps. We also created co-expression maps for other model organisms. Our new web application will allow users to explore and download data from the GeneFriends webserver. Overall, with our latest version of co-expression networks we hope to make GeneFriends unique, powerful and valuable to the scientific community.

## DATA AVAILABILITY

All gene and transcript co-expression maps are available for download at http://www.genefriends.org (https://www.dropbox.com/sh/jz0z3z8fuhx70fx/AACt3CUvyro2cEETVBoWwIrNa?dl=0). Additionally, the code can be found in GitHub (https://github.com/maglab/genefriends_v5).

## Supplementary Material

gkac1031_Supplemental_Files
