# Supplementary material for: GeneFriends: gene co-expression databases and tools for humans and model organisms
Source: Nucleic Acids Res. 2022 Dec 1;51(D1):D145–58. doi: 10.1093/nar/gkac1031 (PMC9825523; doi:10.1093/nar/gkac1031)
Supplement: gkac1031_Supplemental_Files [file gkac1031_supplemental_files.zip › Supplementary_material.pdf]

SUPPLEMENTARY MATERIAL

GeneFriends: Gene co-expression databases and tools for humans and model organisms

(I)LIST OF SUPPLEMENTARY TABLES

Supplementary Table S1. Overlapping of co-expression partners between old and new GeneFriends co-expression database.

Supplementary Table S2. The number of one-to-one orthologs genes between species.

Supplementary Table S3. The number of genes in human and mouse tissue-specific GeneFriends co-expression database.

Supplementary Table S4. The number of tau-based tissue-specific genes in human and mouse.

(II) LIST OF SUPPLEMENTARY FIGURES

Supplementary Figure S1. Number of samples in each tissue for (A) Human tissue-specific GeneFriends co-expression database (B) Mouse tissue-specific GeneFriends co-expression database.

Supplementary Figure S2. The distribution of samples among TCGA and GTEx GeneFriends co-expression database. (A) Distribution of samples among 33 cancer types. (B) Distribution of samples among different tissues in TGCA co-expression database. (C) Distribution of samples among GTEX co-expression database.

Supplementary Figure S3. Distribution of median co-expression values among the different human and mouse tissues.

Supplementary Figure S4. Comparison of KEGG pathway annotations among the top 5% and bottom 5% of the GeneFriends database genes.

Supplementary Figure S5. Comparison of 186 KEGG pathway annotations among top 5% of GeneFriends database genes.

Supplementary Figure S6. (A) Enrichment of cancer driver genes in network modules. The bar chart represents odds ratios calculated from Fisher's exact test. Modules with significant enrichment of cancer driver genes. Asterisks denote significant enrichment of cancer driver genes in modules (adj. p-value < 0.0001). **(B)** Top 10 Gene Ontology (GO) terms related to genes in module 32. (**C**) Top 10 Gene Ontology (GO) terms related to genes in module 90.

Supplementary Figure S7: Screenshot of 'start analysis' step.

Supplementary Figure S8: Screenshot of gene/transcript input, species, data source, object type and tissue

Supplementary Figure S9: Screenshot of gene/transcript present in the database and setting Pearson correlation coefficient threshold.

Supplementary Figure S10: Screenshot of top co-expressed genes (friends).

Supplementary Figure S11: Screenshot of functional annotation of top 5% co-expressed genes using DAVID API

Supplementary Figure S12: Screenshot of network visualization.

(III) GENEFRIENDS TUTORIAL

SUPPLEMENTARY TABLES

Supplementary Table S1. Overlapping of co-expression partners between old and new GeneFriends co-expression database

**Gene name**	**Overlap percentage (new data vs old data)**
BCL6	33
BRCA1	35
CDKN2A	29
FOXO3	24
IGF1R	28
PARP1	40
SIRT1	30
TP53	27
TUG1	25
CDC6	34

Old data = GeneFriends older version by Sipko et al. (2015)

New data = Updated version of GeneFriends

Supplementary Table S2. The number of one-to-one orthologs genes between species

	**Human**	**Mouse**	**Yeast**	**Fruit fly**	**Rat**	**Worm**	**Zebrafish**	**Chicken**	**Cow**
**Human**	-	15741	1228	2970	14894	2513	9088	11827	15398
**Mouse**	15741	-	1311	3150	16107	2652	9490	12195	15221
**Yeast**	1228	1311	-	1436	1252	1441	1236	1259	1255
**Fruit fly**	2970	3150	1436	-	2985	3421	2821	3016	2989
**Rat**	14894	16107	1252	2985	-	2527	9032	11660	14673
**Worm**	2513	2652	1441	3421	2527	-	2369	2542	2510
**Zebrafish**	9088	9490	1236	2821	9032	2369	-	8888	9134
**Chicken**	11827	12195	1259	3016	11660	2542	8888	-	11003
**Cow**	15398	15221	1255	2989	14673	2510	9134	11003	-

Supplementary Table S3. The number of genes in human and mouse tissue-specific GeneFriends co-expression database

**Tissue**	**Human (number of genes)**	**Mouse (no. of genes)**
Adipose	19656	17363
Bone	18971	16615
Bone marrow	25141	18726
Brain	23654	19013
Breast	26198	18608
Esophagus	19623	-
Heart	21622	18901
Intestine	23223	19226
Kidney	21120	20040
Liver	19141	20342
Lung	26497	14293
Lymphoid	-	17949
Muscle	21271	18784
Myeloid	-	17160
Neuron	17887	19460
Ovary	18044	16191
Pancreas	20637	20489
Prostate	26115	-
Retina	21746	19695
Skin	24330	18294
Spleen	18886	19509
Testis	20186	19760
Thymus	-	18797

Supplementary Table S4. The number of tau-based tissue-specific genes in human and mouse.

**Tissue**	**Human (number of genes)**	**Mouse (no. of genes)**
Adipose	2231	3245
Bone	1834	4008
Bone marrow	2744	2710
Brain	4657	4373
Breast	5049	2289
Esophagus	3031	-
Heart	837	1862
Intestine	4209	3922
Kidney	4952	3614
Liver	1617	2173
Lung	4412	3116
Lymphoid	-	2407
Muscle	2275	3467
Myeloid	-	3359
Neuron	2240	2395
Ovary	3427	4359
Pancreas	1457	2143
Prostate	7196	-
Retina	3867	4187
Skin	3004	4378
Spleen	2550	2841
Testis	7536	6258
Thymus	-	4450

SUPPLEMENTARY FIGURES

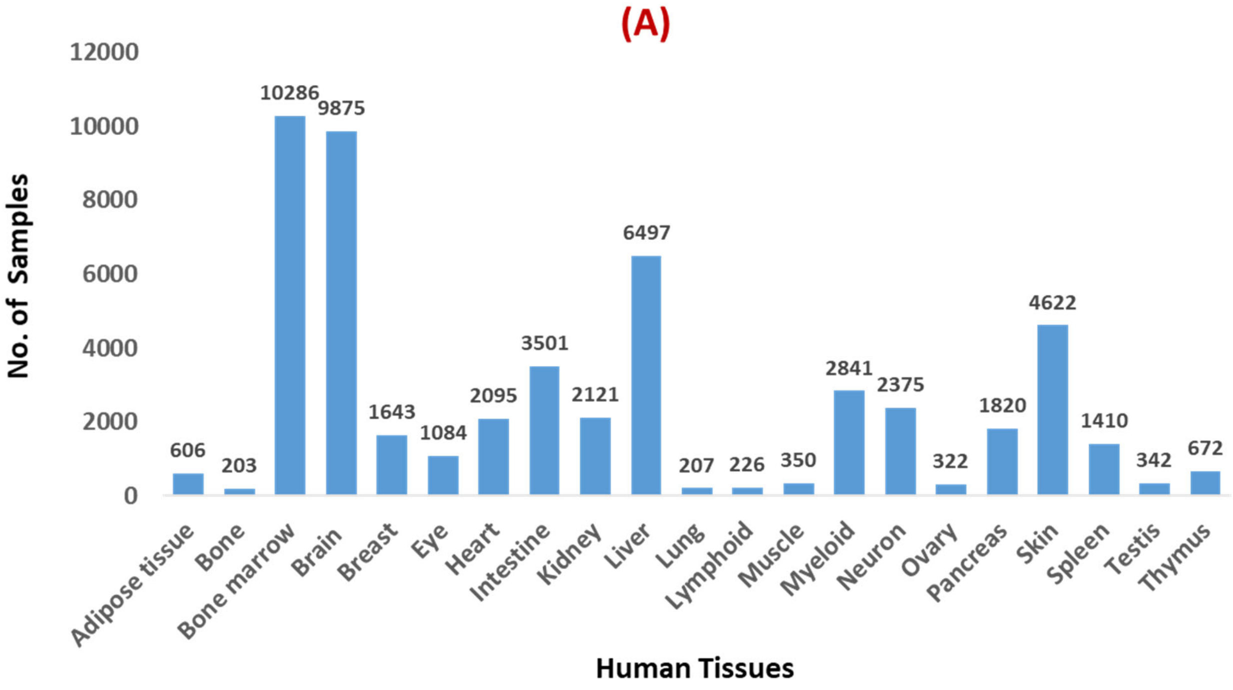

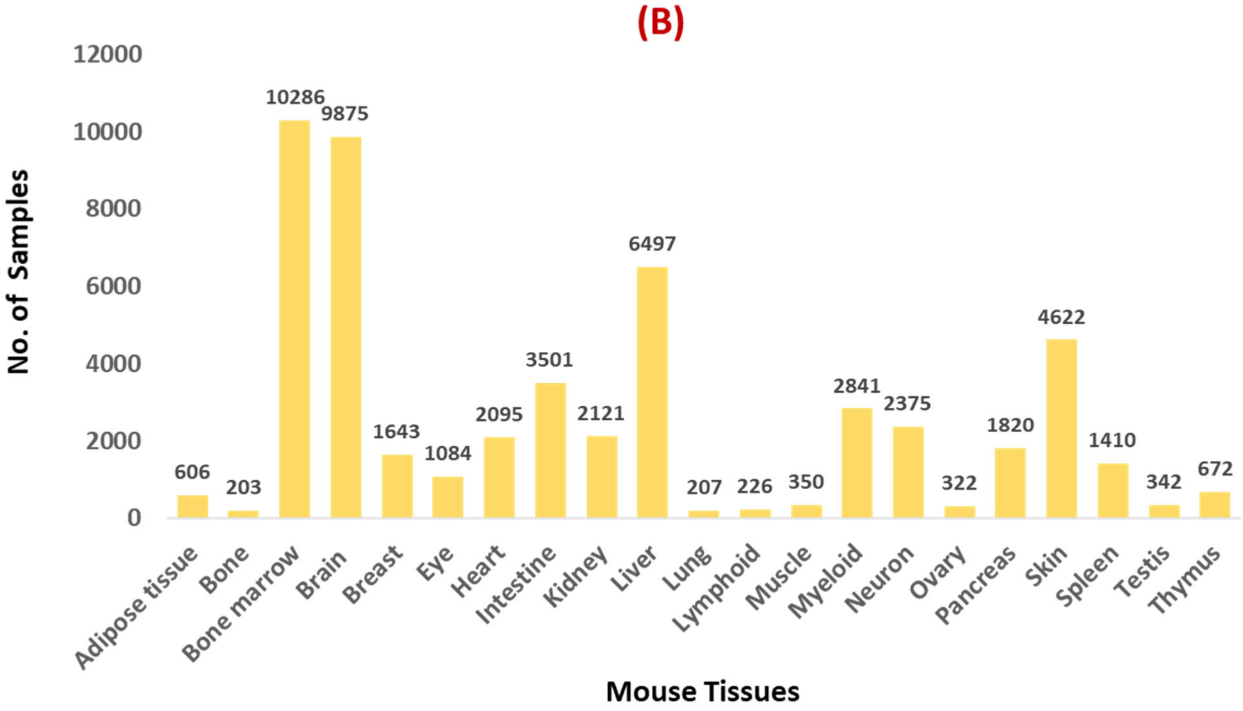

Supplementary Figure S1. Number of samples in each tissue for (A) Human tissue-specific GeneFriends co-expression database (B) Mouse tissue-specific GeneFriends co-expression database.

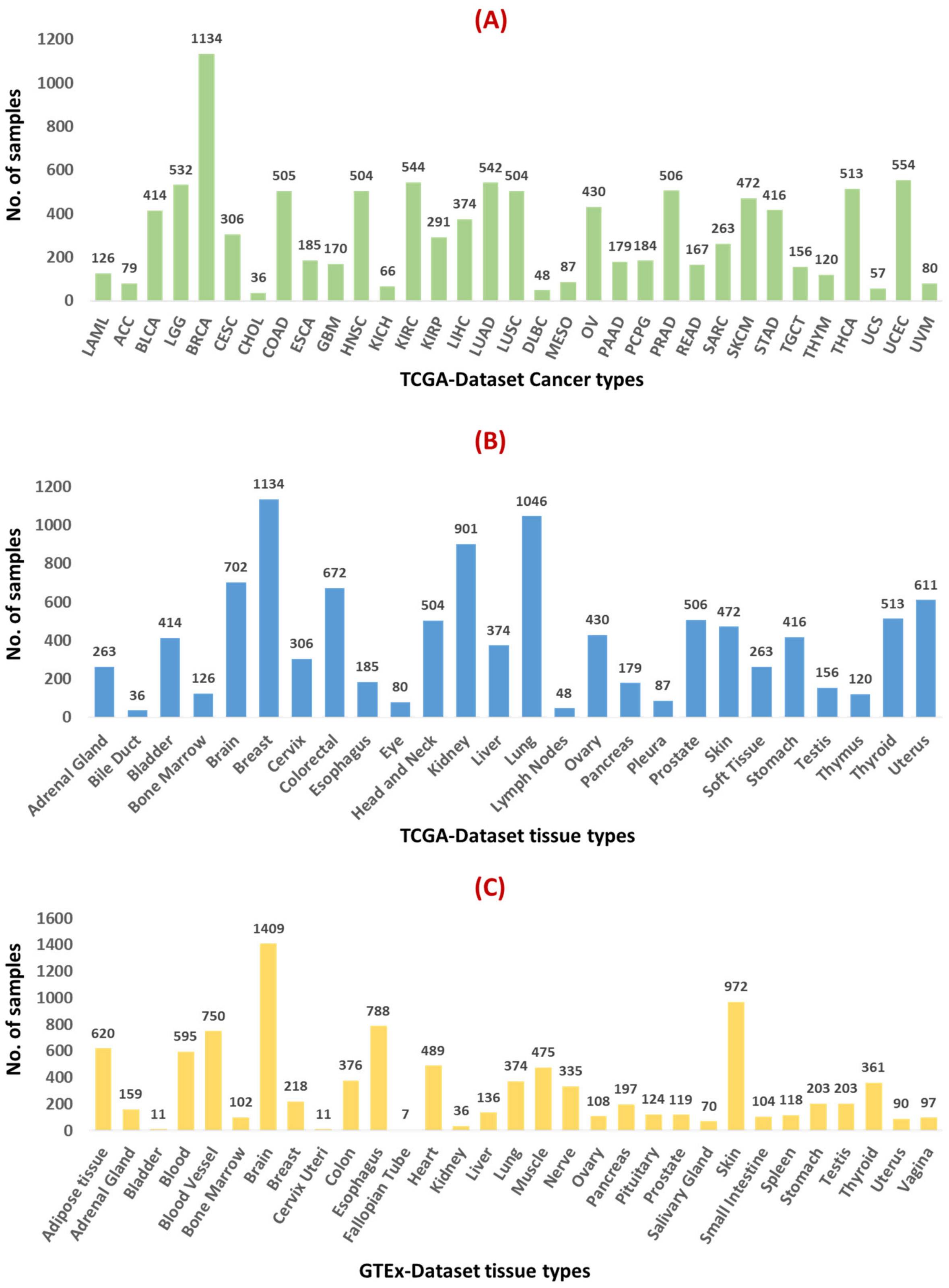

Supplementary Figure S2. The distribution of samples among TCGA and GTEx GeneFriends co-expression database. (A) Distribution of samples among 33 cancer types. (B) Distribution of samples among different tissues in TGCA co-expression database. (C) Distribution of

samples among GTEX co-expression database. LAML=Acute Myeloid Leukemia, ACC=Adrenocortical carcinoma, BLCA=Bladder Urothelial Carcinoma, LGG=Brain Lower Grade Glioma, BRCA=Breast invasive carcinoma, CESC=Cervical squamous cell carcinoma and endocervical adenocarcinoma, CHOL=Cholangiocarcinoma, COAD=Colon adenocarcinoma, ESCA=Esophageal carcinoma, GBM=Glioblastoma multiforme, HNSC=Head and Neck squamous cell carcinoma, KICH=Kidney Chromophobe, KIRC=Kidney renal clear cell carcinoma, KIRP=Kidney renal papillary cell carcinoma, LIHC=Liver hepatocellular carcinoma, LUAD=Lung adenocarcinoma, LUSC=Lung squamous cell carcinoma, DLBC=Lymphoid Neoplasm Diffuse Large B-cell Lymphoma, MESO=Mesothelioma, OV=Ovarian serous cystadenocarcinoma, PAAD=Pancreatic adenocarcinoma, PCPG=Pheochromocytoma and Paraganglioma, PRAD=Prostate adenocarcinoma, READ=Rectum adenocarcinoma, SARC=Sarcoma, SKCM=Skin Cutaneous Melanoma, STAD=Stomach adenocarcinoma, TGCT=Testicular Germ Cell Tumors, THYM=Thymoma, THCA=Thyroid carcinoma, UCS=Uterine Carcinosarcoma, UCEC=Uterine Corpus Endometrial Carcinoma, UVM=Uveal Melanoma

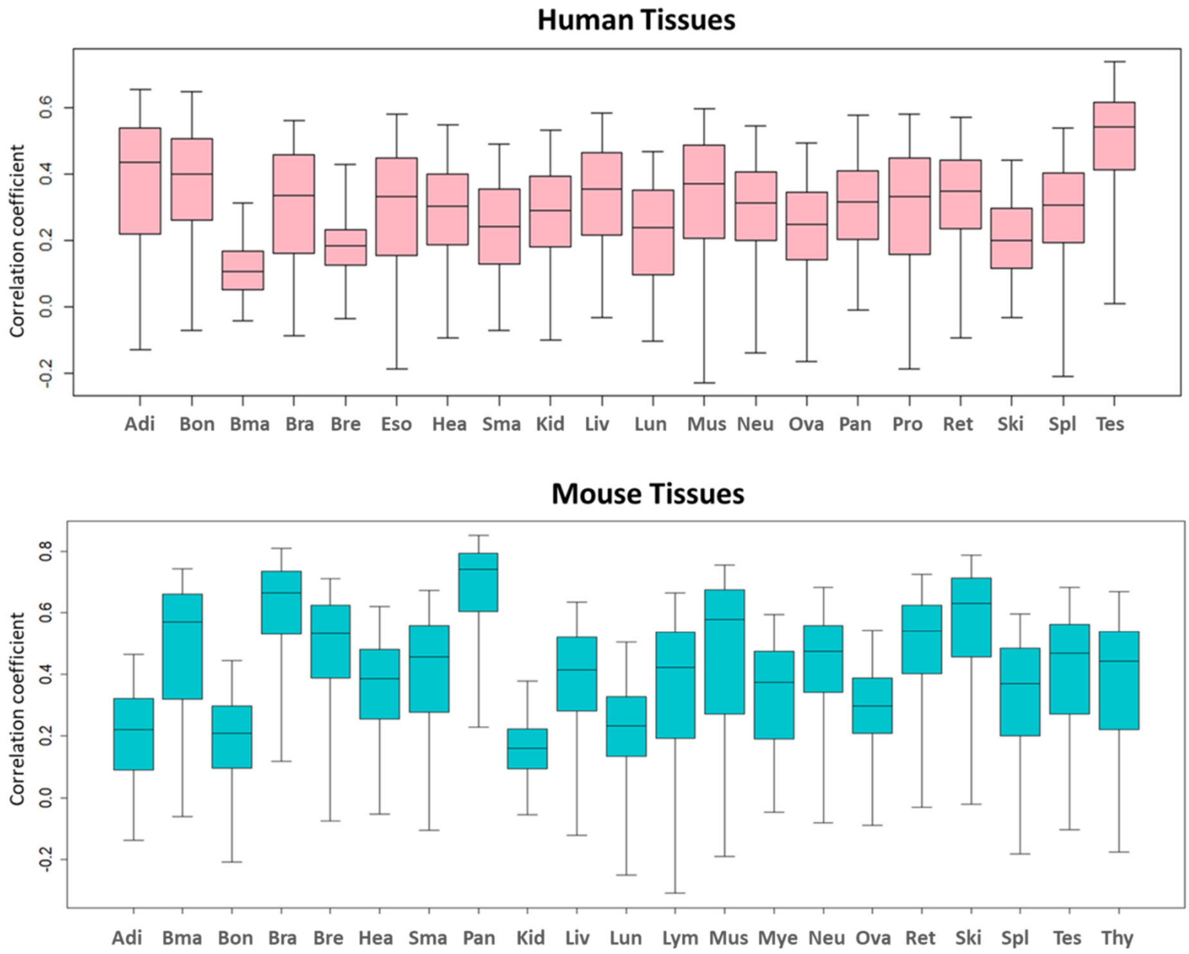

Supplementary Figure S3. Distribution of median co-expression values among the different human and mouse tissues. Adi=Adipose, Bon = Bone, Bma=Bone marrow, Bra=Brain, Bre=Breast, Eso=Esophagus, Hea=Heart, Sma=Small Intestine, Kid=Kidney, Liv=Liver, Lun=Lung, Lym=Lymphoid, Mus=Muscle, Mye=Myeloid, Neu=Neuron, Ova=Ovary, Pan=Pancreas, Pro=Prostate, Ret=Retina, Ski=Skin, Spl=Spleen, Tes=Testis, Thy=Thymus

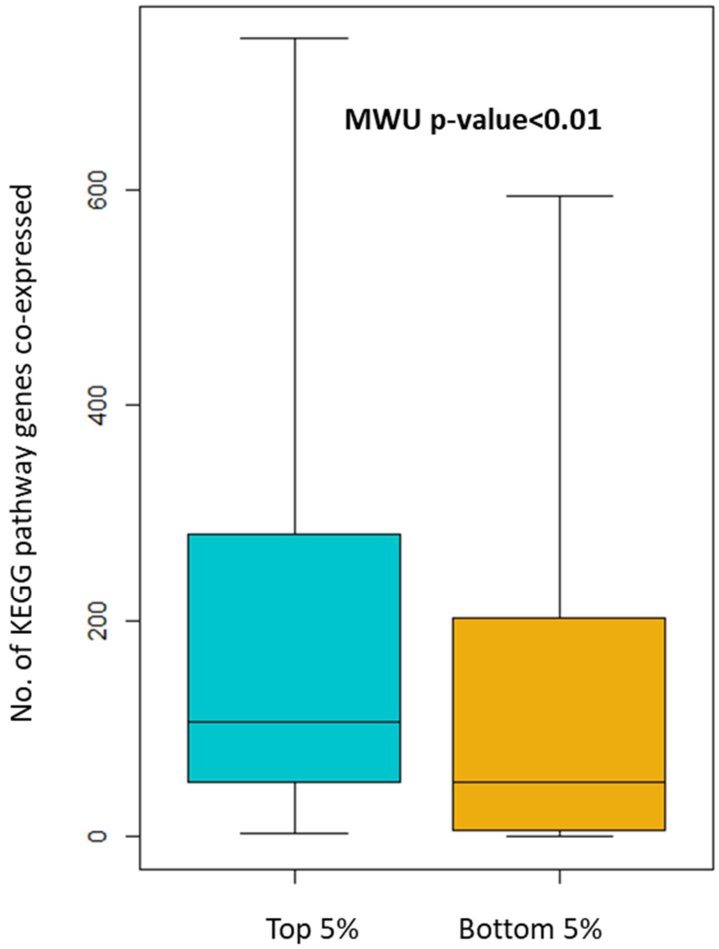

Supplementary Figure S4. Comparison of KEGG pathway annotations among the top 5% and bottom 5% of the GeneFriends database genes.

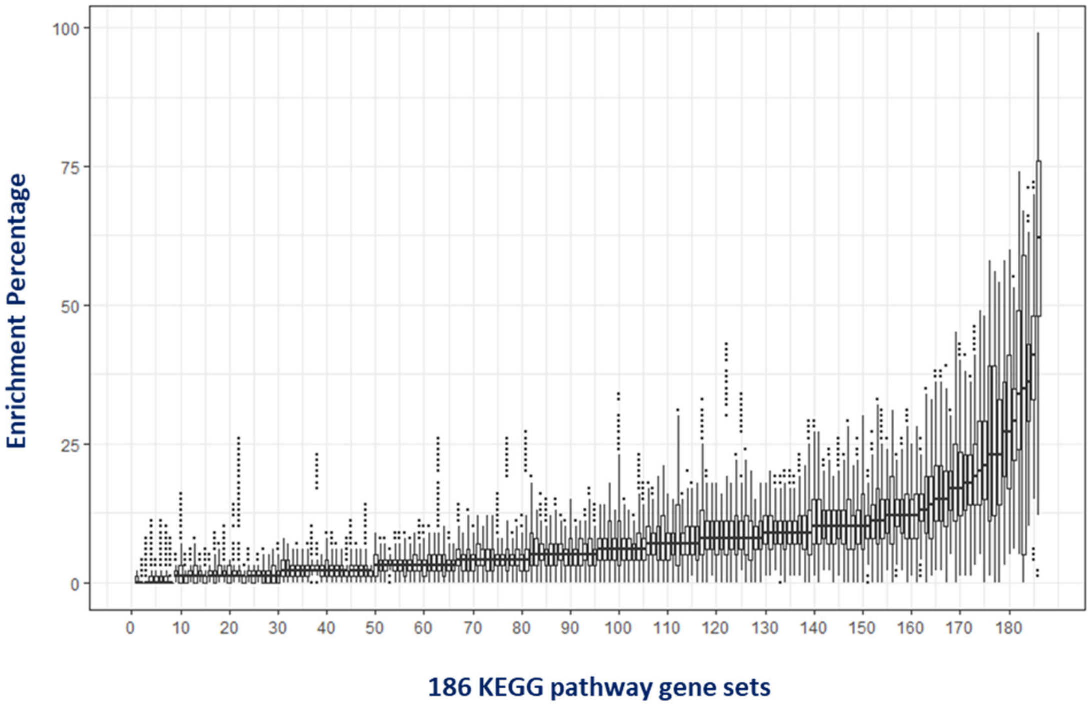

Supplementary Figure S5. Comparison of 186 KEGG pathway annotations among top 5% of GeneFriends database genes.

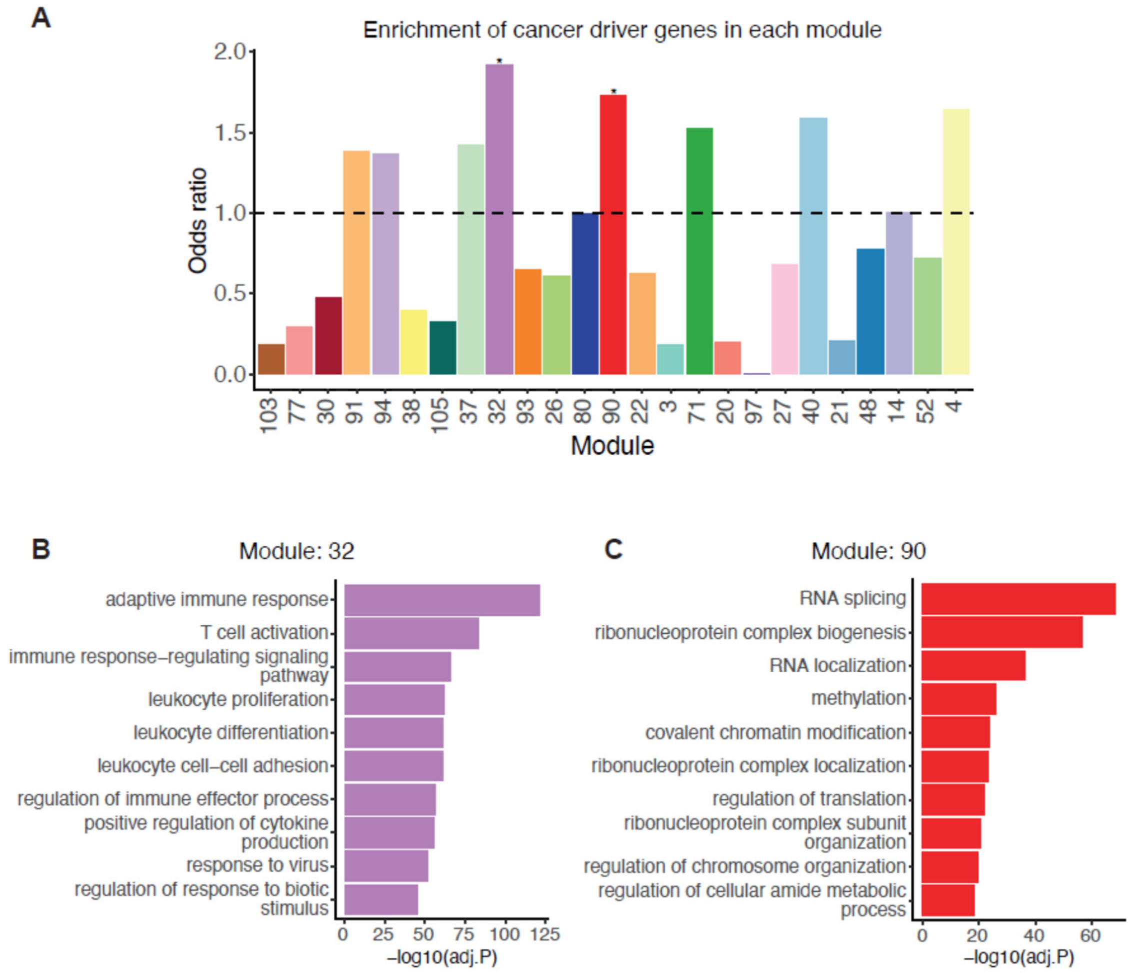

Supplementary Figure S6. (A) Enrichment of cancer driver genes in network modules. The bar chart represents odds ratios calculated from Fisher's exact test. Modules with significant enrichment of cancer driver genes. Asterisks denote significant enrichment of cancer driver genes in modules (adj. p-value < 0.0001). **(B)** Top 10 Gene Ontology (GO) terms related to genes in module 32. (**C**) Top 10 Gene Ontology (GO) terms related to genes in module 90.

<u>**GeneFriends Webserver Tutorial**</u>

In order to use the GeneFriends web application please follow these steps:

Step 1: Start by clicking on the *Start analysis* button/header-link as indicated by the red ellipses shown in supplementary **Figure S7**.

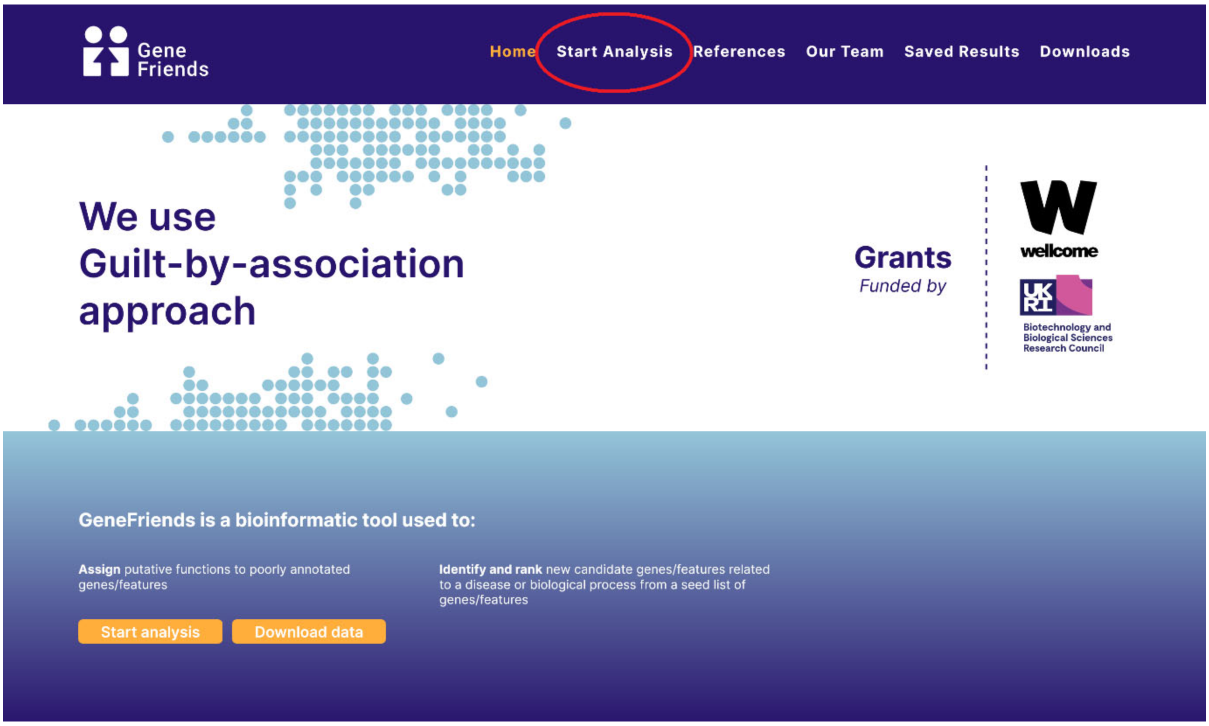

Supplementary Figure S7: Screenshot of 'start analysis' step.

Step 2: Select a species, data source, object type and tissue. Then, input a list of units (Seed list/Identifiers) in the *Genes or Transcripts* field (see supplementary **Figure S8**).

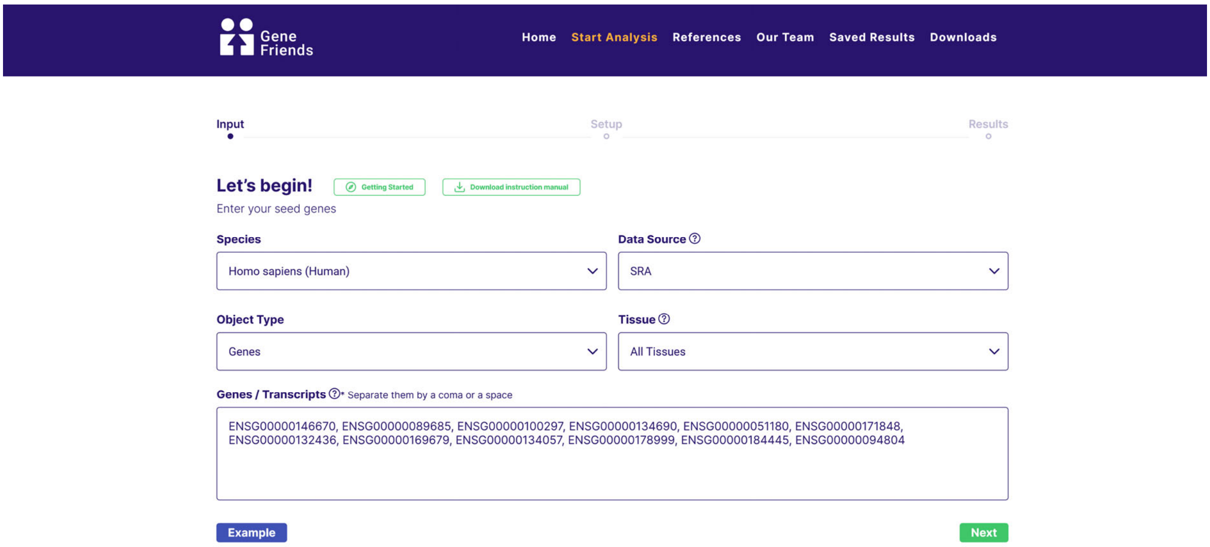

Supplementary Figure S8: Screenshot of gene/transcript input, species, data source, object type and tissue

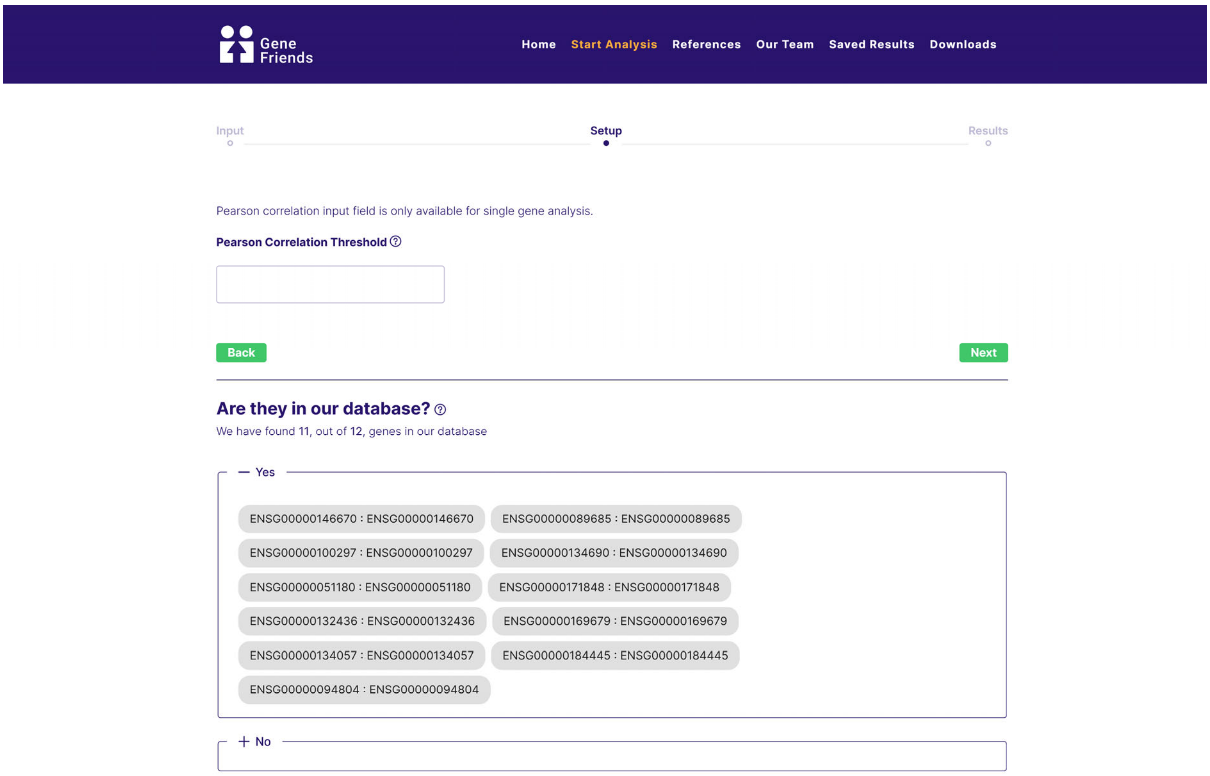

Supplementary Figure S9: Screenshot of gene/transcript present in the database and setting Pearson correlation coefficient threshold.

Step 3: In this step of the process (see **supplementary Figure S9**), the elements of the seed list are looked at in the GeneFriends database. Following this, the list is split into genes which were are present and genes which not present in the database. If just one gene/transcript is found in the database (being because the seed list was composed of a single gene, or because only one matched with our database gene/transcript list), an appropriate Pearson correlation threshold (0.5, by default) must be set before continuing. If more than one gene from the seed list is present in the database, then the Pearson correlation threshold field is deactivated, since it becomes unnecessary for the downstream analysis. Click on the *Next* button to proceed.

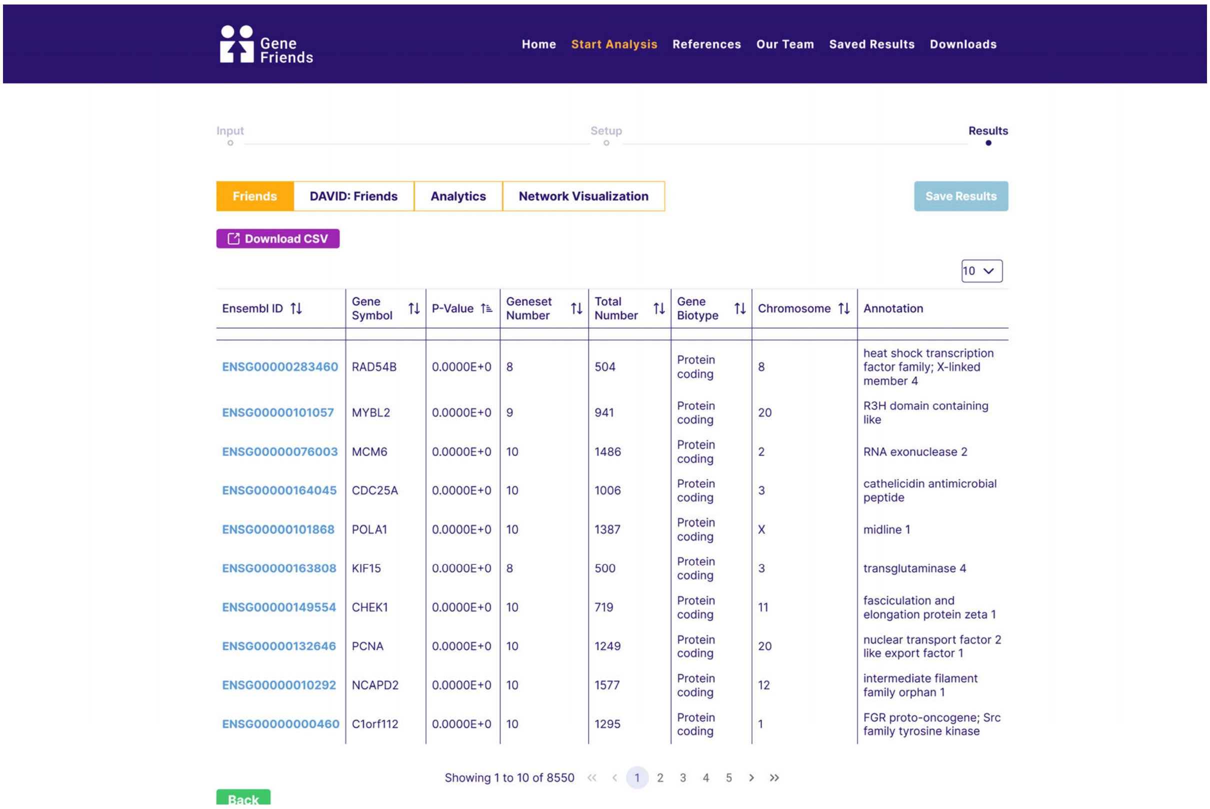

Ensembl ID	Gene Symbol	P-Value	Geneset Number	Total Number	Gene Biotype	Chromosome	Annotation
ENSG00000283460	RAD54B	0.0000E+0	8	504	Protein coding	8	heat shock transcription factor family; X-linked member 4
ENSG00000101057	MYBL2	0.0000E+0	9	941	Protein coding	20	R3H domain containing like
ENSG00000076003	MCM6	0.0000E+0	10	1486	Protein coding	2	RNA exonuclease 2
ENSG00000164045	CDC25A	0.0000E+0	10	1006	Protein coding	3	cathelicidin antimicrobial peptide
ENSG00000101868	POLA1	0.0000E+0	10	1387	Protein coding	X	midline 1
ENSG00000163808	KIF15	0.0000E+0	8	500	Protein coding	3	transglutaminase 4
ENSG00000149554	CHEK1	0.0000E+0	10	719	Protein coding	11	fasciculation and elongation protein zeta 1
ENSG00000132646	PCNA	0.0000E+0	10	1249	Protein coding	20	nuclear transport factor 2 like export factor 1
ENSG00000010292	NCAPD2	0.0000E+0	10	1577	Protein coding	12	intermediate filament family orphan 1
ENSG00000000460	C1orf112	0.0000E+0	10	1295	Protein coding	1	FGR proto-oncogene; Src family tyrosine kinase

Supplementary Figure S10: Screenshot of top co-expressed genes (friends).

Step 4: Finally, all results are shown in this step of the process (see supplementary **Figures S10, S11 and S12**). They are divided into four categories:

1) Friends: View that shows the complete list of friend-associated units to the seed genes group as a whole.
2) DAVID - Friends: View that shows the results after performing the functional annotation clustering analysis on the top 5% (at most 1500) friend-associated units.
3) Analytics: View that shows descriptive statistics to draw insights about the friend-associated units.
4) Network Visualisation: View that shows a visual representation of the coexpression networks of friend-associated units. This is a subnetwork of the complete Pearson co-expression network.

It is also possible to save the obtained results in the browser local database. This can be done by clicking on the *Save Results* button (see figures S10-12). Beware that the saved results will be saved as cache. Therefore, if the cache is cleaned, the data will disappear.

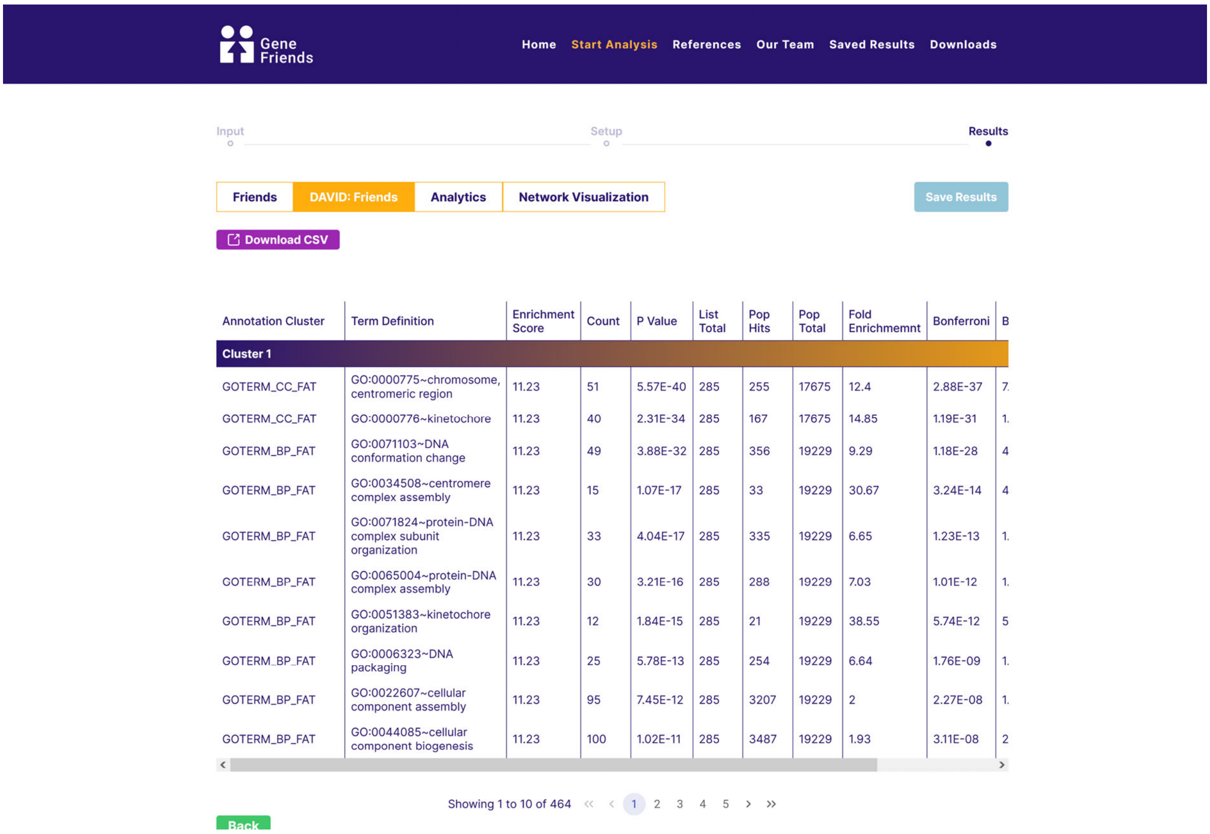

Annotation Cluster	Term Definition	Enrichment Score	Count	P Value	List Total	Pop Hits	Pop Total	Fold Enrichmemnt	Bonferroni	B
Cluster 1										
GOTERM_CC_FAT	GO:0000775~chromosome, centromeric region	11.23	51	5.57E-40	285	255	17675	12.4	2.88E-37	7.
GOTERM_CC_FAT	GO:0000776~kinetochore	11.23	40	2.31E-34	285	167	17675	14.85	1.19E-31	1.
GOTERM_BP_FAT	GO:0071103~DNA conformation change	11.23	49	3.88E-32	285	356	19229	9.29	1.18E-28	4
GOTERM_BP_FAT	GO:0034508~centromere complex assembly	11.23	15	1.07E-17	285	33	19229	30.67	3.24E-14	4
GOTERM_BP_FAT	GO:0071824~protein-DNA complex subunit organization	11.23	33	4.04E-17	285	335	19229	6.65	1.23E-13	1.
GOTERM_BP_FAT	GO:0065004~protein-DNA complex assembly	11.23	30	3.21E-16	285	288	19229	7.03	1.01E-12	1.
GOTERM_BP_FAT	GO:0051383~kinetochore organization	11.23	12	1.84E-15	285	21	19229	38.55	5.74E-12	5
GOTERM_BP_FAT	GO:0006323~DNA packaging	11.23	25	5.78E-13	285	254	19229	6.64	1.76E-09	1.
GOTERM_BP_FAT	GO:0022607~cellular component assembly	11.23	95	7.45E-12	285	3207	19229	2	2.27E-08	1.
GOTERM_BP_FAT	GO:0044085~cellular component biogenesis	11.23	100	1.02E-11	285	3487	19229	1.93	3.11E-08	2

Supplementary Figure S11: Screenshot of functional annotation of top 5% co-expressed genes using DAVID API.

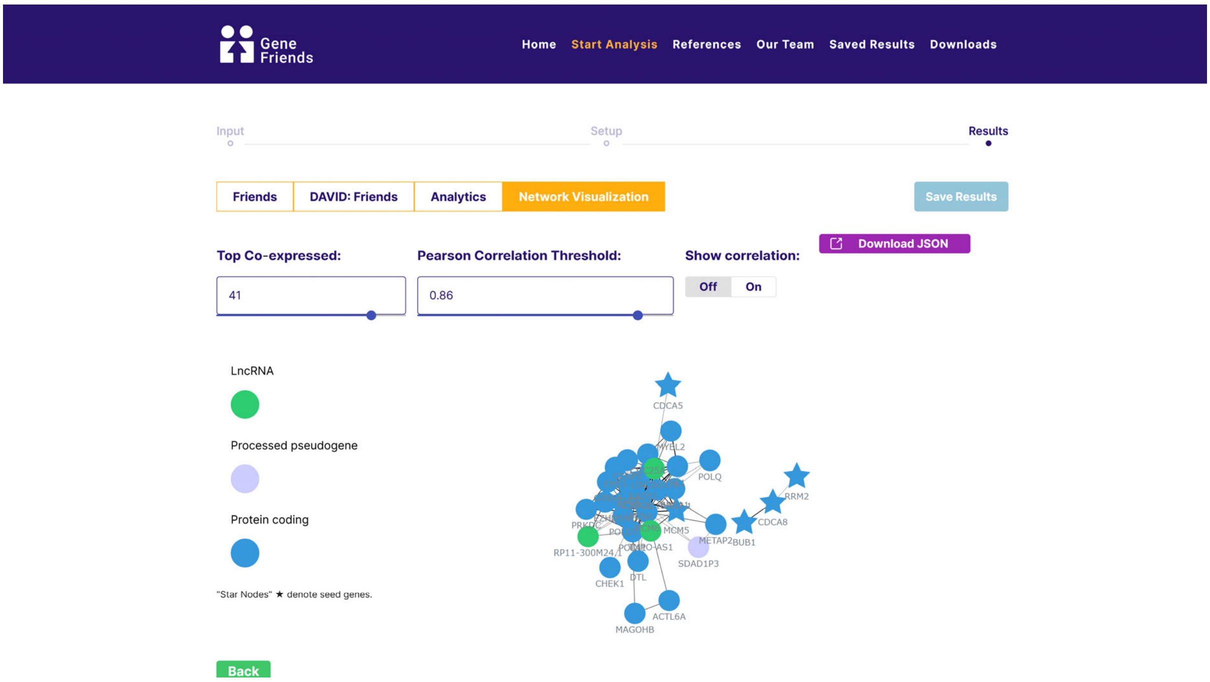

Supplementary Figure S12: Screenshot of network visualization.
